# Scrofuloderma: The Neglected Tuberculosis

**DOI:** 10.1590/0037-8682-0071-2024

**Published:** 2024-05-06

**Authors:** Alan Bittencourt da Silva, Ana Cristina do Amaral Jacques Nacao

**Affiliations:** 1 Hospital Federal de Ipanema, Departamento de Medicina Interna, Rio de Janeiro, RJ, Brasil.; 2 Hospital Federal de Ipanema, Comissão de Controle de Infecção Hospitalar, Rio de Janeiro, RJ, Brasil.

**FIGURE 1:**
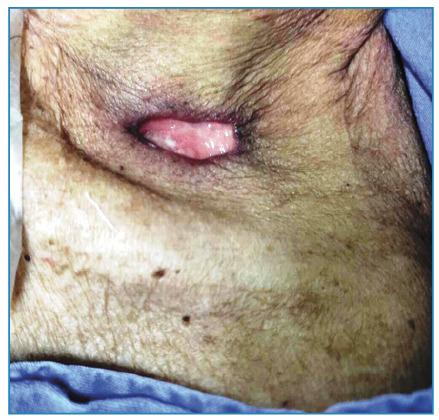
Painless ulcer with a nonexudative base in the left supraclavicular fossa that developed from an enlarged lymph node.

**FIGURE 2:**
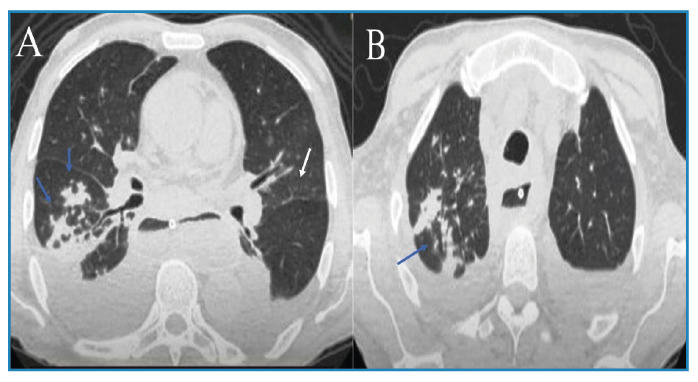
Axial plane thoracic computed tomography images show bilateral pleural effusion, ground-glass opacity in the left lung (white arrow on image A), and a tree-in-bud pattern in the right lung (blue arrows on images A and B).

**FIGURE 3:**
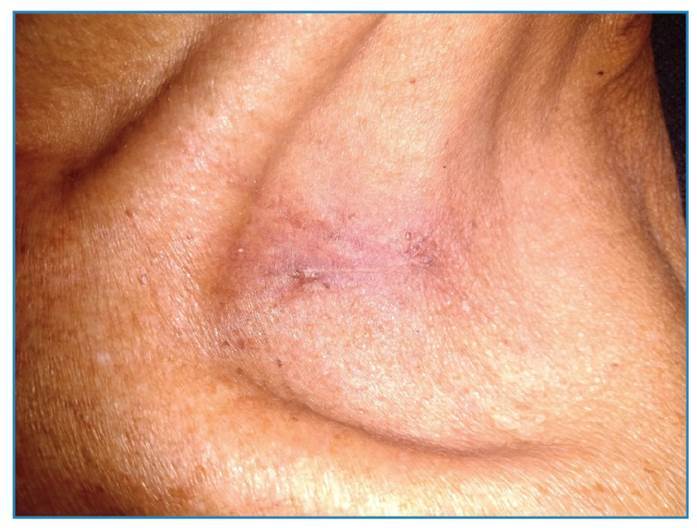
The left supraclavicular ulcer shows complete healing 14 weeks after starting antituberculosis therapy.

A 63-year-old man with achalasia and megaesophagus presented with dry cough, weight loss, fatigue, cervical lymphadenopathy, and a supraclavicular ulcer persisting for five months. Physical examination revealed non-tender cervical lymph nodes without fluctuance or discharge and a painless ulcer in the left supraclavicular fossa with a non-exudative base (Figure 1). Chest computed tomography revealed bilateral pleural effusion, ground-glass opacity in the left lung, and a tree-in-bud pattern in the right lung (Figure 2). The induced sputum samples tested positive for *Mycobacterium tuberculosis*complex through rapid molecular testing (GeneXpert), while yielding negative results on sputum smear microscopy. Standard anti-tuberculosis therapy (two months of intensive treatment followed by four months of continuation) was initiated. Fourteen weeks after initiating therapy, the patient exhibited weight gain, symptom resolution, and complete ulcer healing in the left supraclavicular fossa with no further enlargement of the cervical lymph nodes (Figure 3). Cutaneous tuberculosis cases represents 1-2% of that of extrapulmonary tuberculosis^1^. Scrofuloderma is a type of cutaneous tuberculosis caused by the contiguous spread of *M. tuberculosis* from infected structures such as bones, lymph nodes, and epididymis into the overlying skin. The cervical, supraclavicular, axillary, and inguinal lymph nodes are often involved^1^. Pulmonary tuberculosis is more common in patients with megaesophagus, and most scrofuloderma cases are accompanied by pulmonary tuberculosis^1^^,^^2^. The diagnosis of scrofuloderma was confirmed by complete healing of the ulcer during antituberculosis treatment. Scrofuloderma should always be considered as a differential diagnosis of lymphadenopathy with skin ulcerations, particularly in tuberculosis-endemic areas.
